# Strategies for Restoration of Compromised First Permanent Molars in Children: Challenges and Optimal Timing

**DOI:** 10.3290/j.ohpd.c_2175

**Published:** 2025-08-08

**Authors:** Haojie Yu, Cheng Chen, Qin Shan, Yaoqiong Wang, Mengxin Tian, Qingjing Wang

**Affiliations:** a Haojie Yu Pediatric Dentist, Stomatology Hospital, School of Stomatology, Zhejiang University School of Medicine, Zhejiang Provincial Clinical Research Center for Oral Diseases, Key Laboratory of Oral Biomedical Research of Zhejiang Province, Cancer Center of Zhejiang University, Hangzhou, China. Drafted and revised the manuscript, gave final approval and agreed to be accountable for all aspects of the work.; b Cheng Chen Prosthodontist, Stomatology Hospital, School of Stomatology, Zhejiang University School of Medicine, Zhejiang Provincial Clinical Research Center for Oral Diseases, Key Laboratory of Oral Biomedical Research of Zhejiang Province, Cancer Center of Zhejiang University, Hangzhou, China. Drafted and revised the manuscript, gave final approval and agreed to be accountable for all aspects of the work.; c Qin Shan Pediatric Dentist, Stomatology Hospital, School of Stomatology, Zhejiang University School of Medicine, Zhejiang Provincial Clinical Research Center for Oral Diseases, Key Laboratory of Oral Biomedical Research of Zhejiang Province, Cancer Center of Zhejiang University, Hangzhou, China. Drafted and revised the manuscript, gave final approval and agreed to be accountable for all aspects of the work.; d Yaoqiong Wang Oral and Maxillofacial Radiologist, Stomatology Hospital, School of Stomatology, Zhejiang University School of Medicine, Zhejiang Provincial Clinical Research Center for Oral Diseases, Key Laboratory of Oral Biomedical Research of Zhejiang Province, Cancer Center of Zhejiang University, Hangzhou, China. Acquired and interpreted data, gave final approval and agreed to be accountable for all aspects of the work.; e Mengxin Tian Oral and Maxillofacial Radiologist, Stomatology Hospital, School of Stomatology, Zhejiang University School of Medicine, Zhejiang Provincial Clinical Research Center for Oral Diseases, Key Laboratory of Oral Biomedical Research of Zhejiang Province, Cancer Center of Zhejiang University, Hangzhou, China. Acquired and interpreted the data, gave final approval and agreed to be accountable for all aspects of the work.; f Qingjing Wang Professor, Key Laboratory of Artificial Organs and Computational Medicine of Zhejiang Province, Key Laboratory of Pollution Exposure and Health Intervention of Zhejiang Province, Shulan International Medical College, Zhejiang Shuren University, Hangzhou, China. Study concept and design, gave final approval and agreed to be accountable for all aspects of the work.

**Keywords:** first permanent molar, pediatric dentistry, time factor, tooth restoration.

## Abstract

**Purpose:**

To evaluate restorative strategies for compromised first permanent molars in pediatric patients, with emphasis on determining the optimal timing for intervention.

**Materials and Methods:**

A comprehensive literature search was conducted across four electronic databases: PubMed, ScienceDirect, Scopus, and Web of Science. Among the 127 retrieved articles, 42 studies that met the predefined inclusion criteria were incorporated into the analysis.

**Results:**

Restoration of compromised first permanent molars can be accomplished through both direct and indirect techniques. Standardized protocols for the management of severely compromised first permanent molars are still lacking.

**Conclusions:**

The principal determinants guiding treatment encompass patient cooperation, defect severity, dental developmental stage, pulp status and passive eruption.

**Clinical Relevance:**

High-quality research is required to establish evidence-based guidelines for the restoration of compromised molars in the pediatric population.

A young permanent tooth is characterized as one that has emerged into the oral cavity but has not yet achieved full morphological and structural maturity. The relationship between the maxillary and mandibular first permanent molars is crucial for occlusion classification and plays a significant role in occlusal development.^
5
^ The first permanent molar, which emerges early and is highly susceptible to caries, is frequently mistaken for a deciduous tooth, leading to delayed treatment.^
174
^ Common issues with children’s first permanent molars include caries, pulp and periapical diseases, and structural anomalies such as enamel hypoplasia, dentin dysplasia, fluorosis, and poor mineralization.^
179
^ Long-term defects in these teeth can result in mesial and distal spacing reduction, elongation and displacement of opposing teeth, and diminished masticatory efficiency.^
110
^ When large areas of the tooth are missing and only minimal tooth structure remains, direct resin restorations may be prone to failure due to detachment or fracture of the weakened surrounding tooth structure.^
104
^


Losing the first permanent molar can significantly impact a child’s occlusal relationship, digestive function, and overall development.^
157
^ Since children have not yet achieved full occlusal balance, permanent restorations are challenging.^
5
^ Clinically, transitional restorations are often employed to restore tooth morphology and occlusal function, while avoiding interference with permanent restorations or the eruption of adjacent permanent teeth.^
1
^ These restorations ensure a secure coronal seal to prevent bacterial infection until a permanent restoration can be done in adulthood. Early intervention in restoring defects in the first permanent molar can reduce the risk of poor clinical outcomes.^
18
^ The present review aims to guide the clinical management of tooth defects in children’s first permanent molars.

## Selection of Restorative Methods (Fig 1)

**Fig 1 fig1:**
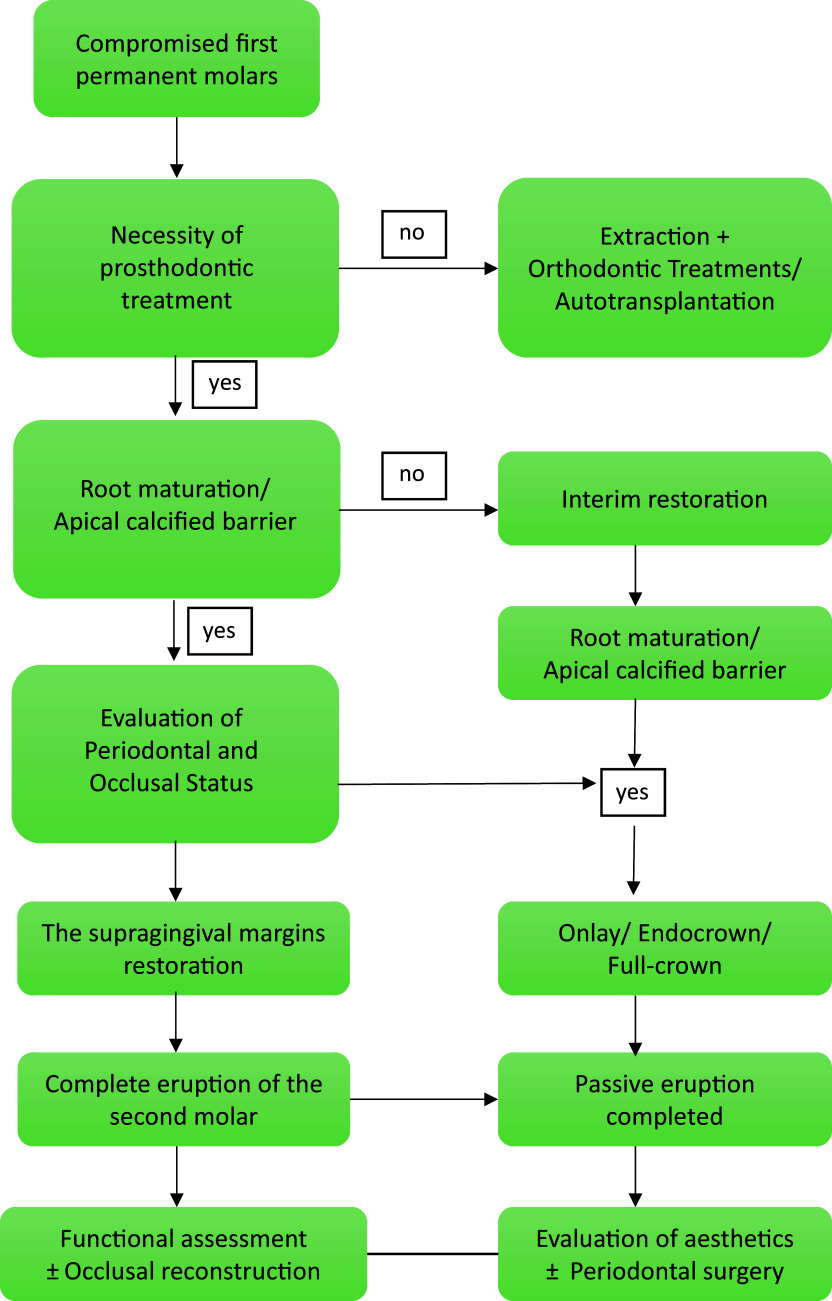
Flowchart for selction of appropriate restorative method.

The choice of restorative method primarily depends on the cooperation, the remaining tooth structure, the structural, chemical, and mechanical properties of both enamel and dentin, the occlusal conditions and the pulp status.^
139
^ Prior to restorative intervention, it is essential to primarily consider the patient’s previous dental treatments, the depth of the cavity, and the condition of the pulp.^
20
^ Ideally, the remaining tooth structure should have a dentin shoulder of at least 1.5 mm in height and 1 mm in thickness.^
111
^ Recent research has indicated that full cusp coverage restorations are necessary when significant structural loss occurs, such as in cases of mesio-occlusal (MO) or disto-occlusal(DO) cavities with axial wall thicknesses less than 2 mm, or in mesio-occlusal-distal (MOD) cavities and cases where structural loss exceeds that observed in MOD cavities.^
22
^ Conversely, when the remaining wall thickness exceeds 2 mm, the reduction in stiffness caused by occlusal access is limited to approximately 5%.^
58
^ Therefore, direct adhesive restorations may be considered a viable alternative to cusp coverage restorations only in these specific scenarios, provided a stable occlusal environment is maintained, with higher success rates reported under such conditions.^
68
^ Alternatively, sufficient root length should be available to allow for crown lengthening or orthodontic methods to extend the clinical crown, ensuring a crown-to-root ratio of at least 1:1 to withstand potential lateral forces.^
97
^ To ensure the restored first permanent molar can perform normal masticatory functions, the patient’s occlusal condition must be assessed.^
98
^ For patients with bruxism or a tight occlusion that generates excessive occlusal forces, full crowns or post-core crowns are recommended.^
166
^ In cases of large defects in the first permanent molars, crown restoration can provide adequate protection.^
165
^


## Restorative Techniques

### Literature Search Strategy

The inclusion criteria for this study were as follows: articles published before 21 March 2025 and written in English; reviews, case reports, book chapters, meta-analyses, randomized controlled trials, clinical trials, and prospective and retrospective cohort observational studies were included, while articles without full text available were excluded. A systematic search was carried out on 21 March 2025 in PubMed, ScienceDirect, Scopus, and Web of Science using different combinations of the following search terms and their synonyms: “first permanent molars,” “tooth defect,” “restoration,” “restorative techniques,” “restorative treatment,” “children,” “pediatric,” “composite restoration,” “molar incisor hypomineralization,” “enamel hypoplasia,” “composite resin,” “preformed metal crown,” “inlays,” “all-ceramic crowns,” “onlays,” “post and core,” “implant timing,” and “treatment timing.”

### Direct Glass-Ionomer Restoration

Compromised first permanent molars (cFPMs) presenting with mild to moderate defects, where carious lesions are limited to enamel or the outer one-third of dentin, can be restored using minimally invasive (MI) techniques.^
7
^ The use of glass-ionomer cement (GIC) in caries management represents a promising treatment strategy aligned with MI principles.^
90
^ Conventional GIC releases fluoride ions in a sustained manner, thereby affording superior anticaries efficacy relative to other restorative materials by inhibiting demineralization, promoting remineralization, and suppressing bacterial proliferation.^
20,196
^ GIC exhibits negligible volumetric shrinkage and expansion change, with a coefficient of thermal expansion matching that of natural tooth structure.^
88
^ Moreover, in comparison to resin-based adhesive systems, GIC induces less pulpal irritation and affords excellent biocompatibility.^
91,107
^ GIC bonds directly to dental hard tissues without the requirement for supplemental bonding agents or pre-application acid etching, thereby streamlining clinical procedures and reducing operative time.^
169
^ Compared with resin‑based materials, GIC exhibits superior hydrophilicity, resulting in low technique sensitivity in moist environments.^
8
^ These distinctive properties facilitate GIC eminently suitable for a broad spectrum of applications. For partially erupted or sensitive cFPMs, the application of conventional GIC using the “finger-press” technique for fissure sealing serves as an effective transitional treatment.^
81
^ This approach effectively alleviates patient discomfort, making it particularly suitable for pediatric patient who exhibit limited cooperation.^
183
^


Nonetheless, GIC is characterized by several drawbacks, including lower compressive and flexural strengths, compromised aesthetic properties, and limited antimicrobial efficacy.^
144
^ The limited mechanical strength of GIC restricts its application in loadbearing regions.^
47,77,155
^ In regions subject to occlusal wear, the limited wear resistance of GIC curtails the long-term durability of restorations.^
206
^ The extent to which fluoride augments the antimicrobial properties of GIC remains marginal, particularly following the completion of the setting reaction.^
16,195
^ Clinical investigations have identified secondary caries as the principal factor leading to the failure of GIC restorations.^
193
^ Accordingly, various modified GIC formulations have been developed to mitigate these deficiencies.^
199
^


High-viscosity GIC (HVGIC), distinguished by a high powder:liquid ratio, has emerged as the preferred material for atraumatic restorative treatment (ART), attributable to its superior mechanical performance, improved margin sealing, and long-term durability.^
101
^ Resin-modified GIC (RMGIC) presents superior esthetic integration and mechanical performance, facilitating the restoration of occlusal cavities and cervical defects in permanent dentition, and proving particularly advantageous in clinical scenarios where optimal isolation is unattainable.^
21,24,93
^ Nevertheless, RMGIC exhibits increased polymerization shrinkage and diminished capacity for fluoride release.^
109,133,203
^


Silver diamine fluoride (SDF) inhibits the development of cariogenic biofilms and enhances acid resistance by promoting dentin remineralization, preventing collagen degradation, and facilitating the transformation of hydroxyapatite into fluorapatite.^
38,85,130,131,205
^ Furthermore, the application of 38% SDF prior to GIC not only preserves bond strength but also optimizes antimicrobial outcomes.^
105,132,147,198,205
^ SDF serves as a fluoride reservoir, promoting sustained fluoride release through ion exchange by substituting hydroxyl ions within the GIC matrix.^
79,106,145
^ Nevertheless, further investigations are required to validate its longterm efficacy in clinical practice.^
89,92
^


### Direct Composite Restoration

Direct composite restorations offer several advantages, such as preserving tooth structure, ease of retreatment, time efficiency, and cost-effectiveness.^
29
^ They are especially suitable for minor tooth defects, such as pit and fissure or proximal cavities, in patients with a low risk of caries.^
54
^ When appropriate materials are properly utilized, a minimum of 60% of resin composite restorations exhibit durability exceeding ten years.^
117
^ In cases of minimal pit-and-fissure caries, the affected tooth structure can be precisely removed and subsequently restored, thereby eliminating the need for the traditional “extension for prevention” approach.^
24
^ Nonetheless, there are notable drawbacks (recurrent caries, marginal discoloration, composite wear, retention failure and fracture of restorations) to consider.^
121
^ Achieving proper isolation in the distal areas of young permanent molars can be challenging, often resulting in decreased bonding strength and frequent detachment of the filling.^
180
^ Moreover, composite resins may undergo polymerization shrinkage, which can lead to marginal microleakage.^
82
^ Inadequate shaping or excessive polishing of proximal areas may create gaps susceptible to recurrent caries.^
181
^ Properly shaping the occlusal surface and maintaining the occlusal-gingival height can also be difficult, and occlusal wear may reduce chewing efficiency.^
152
^ Furthermore, direct composite restorations do not offer substantial protection to the tooth structure, which could result in fractures under stress.^
143
^ The primary causes of failure include recurrent caries, fracture of restoration, postoperative sensitivity, marginal staining and aesthetic deterioration.^
163
^ Additionally, younger age and a higher number of restored surfaces are associated with a shorter lifespan of the restoration.^
52
^ Despite these issues, direct composite restoration remains a preferred minimally invasive option when there is sufficient remaining tooth structure to provide retention and resistance.^
43
^ This approach can serve as a provisional measure before definitive treatment in adulthood, while also preventing the risks of pulp exposure and the need for crown preparation in younger patients.^
4,125
^


### Composite Resin Inlays

Indirect restorations offer several advantages in terms of anatomical form and reinforcement of a tooth compromised by fracture.^
140
^ In pediatric dentistry, indirect composite resin restorations are particularly beneficial due to their abbreviated treatment duration, which enhances patient satisfaction by alleviating procedural anxiety and discomfort.^
127
^ Composite resin inlays, used as semi-permanent or permanent restorations, exhibit superior properties to conventional light-cured resins, including better thermal expansion coefficients, compressive strength, and wear resistance.^
100,188
^ These inlays are beneficial because they have moderate hardness, which prevents excessive wear on opposing teeth and helps establish a normal occlusal relationship in children.^
60
^ Physiological function is achieved by covering the entire cusp, thereby reducing the risk of tooth fractures.^
197
^ Additionally, composite resin inlays provide a good anatomical shape and proximal contact, promoting periodontal health.^
154
^ The biological compatibility of composite resin inlays is excellent, and their margin lines are easily adjustable and situated in areas that facilitate self-cleaning.^
171
^ Their optical properties ensure color harmony with surrounding teeth, and microleakage is minimized due to minimal polymerization shrinkage in the thin adhesive layer.^
84
^ Compared to full crowns, composite resin inlays require minimal tooth reduction, and if the original restoration is damaged, it can be easily repaired with new composite resin, making it suitable for physiological needs during occlusal development in children.^
136
^ Drawbacks of composite resin inlays include long margin lines that require high bonding standards, susceptibility to staining, fracture and reliance on the precision and polish of the margins.^
69
^ Based on the severity of the defect, restorations can be placed supra-gingivally, sub-gingivally, or intra-gingivally with differing edge smoothness, which may have long-term implications for periodontal tissues.^
55
^ Oral health education and guidance should be intensified for children at high risk of recurrent caries.

### Ceramic Inlays

Studies indicate that indirect restorations, such as inlays and crowns, significantly reduce marginal microleakage or marginal gap formation compared to direct composite restorations.^
43,49
^ They also exhibit a lower failure rate due to recurrent caries and offer greater protection against root fractures following endodontic treatment.^
36
^ Ceramic materials used in these restorations, consist of resin matrix and ceramic particles that closely mimic the mechanical properties of natural teeth, including elastic modulus, compressive strength, flexural strength, and wear rate.^
96
^ Nanoceramic resin crowns offer an advantageous restorative option for young permanent teeth, addressing not only dentin irregularities (DI) but also extensive carious lesions, decalcifications, enamel defects, and challenges associated with humidity control during dental procedures.^
32,71,156
^ This material, comprising 80% nanoceramic particles, exhibits high fracture resistance, excellent bending strength, high elasticity, and aesthetic characteristics comparable to those of natural teeth.^
141
^ Fracture of the restoration is the most common technical complication, followed by loss of retention and fragmentation.^
122
^ Increased occlusal wear and the loss of detailed anatomical morphology in resin-based composite (RBC) restorations are the most commonly detectable forms of damage, indicating a decline in their mechanical wear resistance.^
61
^ Lithium disilicate demonstrates enhanced aesthetic performance over composite resins, along with superior aging resistance and a reduced plaque retention rate.^
122
^ The indirect fabrication process sidesteps polymerization temperature and shrinkage issues, resulting in dense, biocompatible restorations with good buffering capacity.^
160
^ These materials, in terms of anatomic form, offer excellent wear resistance, which helps maintain occlusal space and supports periodontal health through effective polishing.^
23
^ Minimally invasive tooth preparation allows for high-strength cusp coverage, safeguarding the tooth structure and ensuring that gingival margins are preserved for optimal periodontal health.^
30
^ For teeth with extensive caries or pulp and periapical diseases, high inlays and pulp chamber-retained crowns are recommended to preserve as much of the natural tooth structure as possible.^
128
^ When utilizing ceramic restorations, it is essential to consider the shorter crowns of young permanent teeth, the relatively larger pulp chambers in vital teeth, and the varying levels of cooperation among pediatric patients.^
88
^ Decision-making was primarily influenced by cavity factors, including the thickness of the residual cusp wall (interaxial dentin), the presence and thickness of proximal ridges, the depth of the cavity, and the presence or absence of the pulp chamber roof.^
76
^ Class II, two- or three-surface inlays were recommended in the following cases: as a replacement for amalgam or old RBC fillings where the buccal and lingual walls remained intact and were thicker than 2.5 mm; after caries removal, with buccal and lingual walls intact and thicker than 2.5 mm, but with an excessively wide isthmus; for multiple medium-sized cavities in the same quadrant; or as an alternative to medium-sized direct resin composite restorations to address their limitations.^
122
^ Onlays were indicated in situations where the cusp thickness was less than 2.0 mm with a cusp height under 4.0 mm, or less than 2.5 mm with a cusp height greater than 4.0 mm; when signs such as cracks or wide attrition facets indicated traumatic overload on the relatively thick cusp that defined the cavity; or for root canal-treated teeth with one missing marginal ridge, where the other ridge remained intact and both cusps were over 2.5 mm thick.^
122
^ Overlays were recommended for root canal-treated teeth with MOD cavities or axial walls less than 2 mm thick, or when an intracoronal restoration was required with an additional need for increasing the vertical occlusal dimension (VDO).^
122
^ For molars undergoing root canal treatment in late mixed dentition or early permanent dentition, both partial and full-coverage inlays, as well as pulp chamber-retained crowns, are viable options.^
46
^ The incorporation of auxiliary behavioral management methods, such as nitrous oxide sedation and intraoral scanning, may broaden its applicability across a wider range of clinical scenarios.^
45
^ In the posterior regions of the dentition, the application of bonded partial crowns—alternatively known as overlays or occlusal veneers—offers an optimal equilibrium between comprehensive coverage of the tooth apex, conservation of dental tissue, and aesthetic enhancement, provided that sufficient tooth structure remains.^
118
^ Employing more conservative preparation techniques that preserve the existing enamel markedly improves the predictability and success rates of bonded restorations.^
190
^ Occlusal veneers and pulp-retained crowns facilitate the provision of new cusp-covering restorations without necessitating extensive reduction of the tooth’s axial surfaces or subgingival margins, thereby effectively addressing associated severe dental defects.^
118
^ In cases of significant tooth structure loss in young permanent molars, leading to diminished cusp support, the application of cusp-covering restorations provides a viable and aesthetically advantageous solution, offering both structural reinforcement and improved aesthetic outcomes.^
35,56,108
^ Plaque accumulation can result from various factors, including the patient’s oral hygiene practices, the composition of their bacterial flora, and the quality of periodontal maintenance.^
73,74
^ Materials such as lithium disilicate and Polymer-Infiltrated Ceramic Network (PICN) demonstrate superior elastic moduli relative to machinable composite materials, thereby exhibiting enhanced mechanical performance.^
17
^ These advanced materials are highly recommended for use in partial crowns for endodontically treated teeth (ETT) to optimize the bonding interface, facilitate effective force transmission to the underlying tooth structure, and ensure the long-term durability and reliability of the restorative outcome.^
34,73,128
^ Furthermore, this restorative approach presents several disadvantages, including the removal of additional dental tissue, increased difficulty in repairing, and higher associated costs.^
122
^ The most frequent failures for both Lithium disilicate and RBC restorations were bulk fracture with broken onlay cusp, and minor chip fracture of the restoration or tooth, endodontic complication, recurrent caries and loss of retention.^
122
^ Occlusal stresses associated with bruxism mildly compromise restoration integrity, increasing the risk of fracture, while poor oral hygiene further contributes to the occurrence of marginal staining.^
187
^ Using digital oral scanning combined with chair-side computer-aided design and manufacturing (CAD/CAM), resin milling produces restorations without polymerization shrinkage, ensuring excellent biological compatibility, wear resistance, comfort-oriented treatment and precision in pediatric patients.^
46,208
^ This report presents an interdisciplinary treatment strategy for transitioning from mixed to permanent dentition using CAD/CAM technology, specifically applied to preformed crowns in a 10-year-old child with severe amelogenesis imperfecta (AI) and maxillofacial deformities. This approach simplifies the treatment process, reduces chairside operation time, and requires minimal tooth preparation for the transitional full crown restoration.^
135
^ Teeth with deep subgingival margins present significant challenges for integration into digital systems using intraoral scanning technology.^
201
^ The bonding system and pulp chamber retention enhance the bond strength between the inlay and the tooth, providing good retention and shear resistance.^
95
^


### Preformed Metal Crowns

When the remaining tooth structure is insufficient to provide adequate resistance, full-coverage restorations can help mitigate the risk of tooth fractures and less recurrent caries.^
126,168
^ Preformed metal crowns offer robust mechanical retention and restore occlusal function, making them effective semi-permanent solutions for first permanent molars.^
48,186
^ Stainless steel crowns (SSCs) are prefabricated dental restorations specifically designed for the rehabilitation of individual teeth.^
189
^ According to the Guideline on Pediatric Restorative Dentistry, the utilization of SSCs is recommended for teeth that have multiple-surface caries or undergone pulp therapy, particularly in patients with an increased risk of dental caries due to factors such as age, behavioral habits, or prior medical history.^
121
^ They are particularly useful in orthodontic planning where extraction is anticipated, as they help maintain space until the optimal time for extraction.^
148
^ However, it is crucial to prepare enough space distally if the second molar has not yet erupted. Preformed metal crowns require occlusal adjustments post-restoration, with high points gradually wearing down to establish a stable occlusal relationship.^
200
^ In cases where severe disease affects occlusal relationships, adjustments may be necessary before placing the crown to correct any issues impacting normal maxillofacial development.^
53
^ These crowns necessitate less tooth preparation compared to high inlays, which is advantageous for future permanent restorations.^
189
^ They are also more cost-effective, less technique-sensitive, and require shorter chair time, making them suitable for children who are intolerant of lengthy procedures.^
9
^ However, preformed metal crowns can have drawbacks, such as risks of detachment, crown perforation, marginal adaptation, unaesthetic appearance impaction of an adjacent second permanent molar and potential metal allergies.^
37
^ While they generally have lower detachment rates and better anatomical shape and proximal contact compared to resin fillings, the gingival margins under these crowns can accumulate plaque, increasing the risk of gingivitis and proximal caries.^
37,94
^ Therefore, proper oral hygiene guidance and monitoring for periodontal issues or effects on the eruption of the second molar are essential.^
119
^ The longevity of preformed metal crowns can be age-dependent, with failure rates potentially increasing with age due to greater occlusal forces and defect size.^
59,116
^ Preformed metal crowns are often used as transitional restorations for cases involving incomplete root development or root canal treatment.^
59
^ The immediate post-operative placement quality and the loss of the proximal wall plays a crucial role in the long-term success of the restoration.^
37
^ The placement of the gingival margin of stainless steel crowns exerts only a minimal influence on patients’periodontal health indicators.^
102
^ Due to the preformed shape and size of SSCs, achieving optimal marginal fit can be challenging.^
189
^ Permanent SSCs on molars may compromise periodontal health if the crown contour is excessive, the marginal fit is inadequate, or residual cement remains in contact with the gingival sulcus, as all of these factors are associated with plaque accumulation.^
186
^ The placement of SSCs subgingivally or inadequate contour of the crown margins may also pose a risk of damaging the biologic width of the periodontal attachment.^
116,177
^ Furthermore, restoring the normal morphology of severely compromised first permanent molars may be more effective in reducing plaque accumulation.^
114
^ Clinicians may avoid using SSCs in young permanent molars undergoing vital pulp therapy (VPT) and instead recommend the use of direct bonded restorations as a preferable alternative.^
37
^ In studies addressing the treatment of molars affected by molar-incisor hypomineralization (MIH) with preformed metal crowns (PMC), a short-term increase in periodontal pocket depth has been observed.^
48
^ Furthermore, the invasiveness and potential consequences of traditional techniques, such as tooth preparation, should be carefully considered, including the reduction of tooth structure available for bonding and the retention of future restorations.^
116
^ Trimming, cutting, and crimping SSCs have been identified as significant risk factors for plaque accumulation on these restorations.^
129
^ The deep-bite cases, progressive resorption of the root, bruxism or excessive tooth wear are among their contraindications.^
31
^ Consequently, the occlusal functional examination would be incorporated into the preoperative assessment.^
128
^


### All-Ceramic Crowns

All-ceramic crowns offer adequate strength to endure functional loads, while simultaneously ensuring optimal aesthetic outcomes.^
115,124,125
^ All-ceramic materials and bonding techniques provide new solutions for permanent crown restorations in young permanent teeth with extensive defects.^
125
^ The success rate of the procedure seems to correlate positively with the amount of remaining dental wall structure.^
172
^ Specifically, when all four walls of the tooth remain intact, the success rate can reach as high as 100%.^
12
^ In a prospective randomized controlled study on ceramic crown treatment for patients with severe enamel hypoplasia,^
119
^ Procera crowns and 108 IPS e.max Press crowns were placed in patients aged 11 to 22 years.^
153
^ After a 5.5-year follow-up, 95% of the crowns were rated as excellent or acceptable in quality, while 4% required adjustments due to poor marginal integrity.^
126
^ Recent research suggests that passive eruption during adolescence does not affect the aesthetic outcomes of all-ceramic crowns.^
126
^ Full-coverage prefabricated zirconia crown represents a durable restorative approach, capable of withstanding several years of service by ensuring the essential seal required to preserve pulp vitality, resisting escalating masticatory forces, and effectively adapting to growth and occlusal development.^
10
^ Nevertheless, physiological passive eruption may result in the formation of supragingival margins over time.66 Compared to preformed metal crowns, minimally invasive preparation with all-ceramic materials offers the advantage of better preserving tooth structure.^
204
^ In-vitro studies have demonstrated that monolithic zirconia full crowns possess superior post-fatigue fracture resistance when utilized in ETT.^
80
^ The exceptional mechanical properties and high machinability of monolithic zirconia enable the fabrication of crowns with significantly reduced thicknesses.^
44
^ This conservative preparation technique facilitates the preservation of a greater extent of tooth structure, which is imperative for the long-term stability and integrity of restorations in ETT. Zirconia exhibits high biocompatibility and features a polished, smooth surface that reduces plaque accumulation, thereby minimizing gingival irritation.^
33,65
^ Additionally, restorations at the gingival level help maintain periodontal health.^
170
^ This approach ensures that the restoration not only restores function but also integrates well with the natural development of the tooth and surrounding tissues. Studies have demonstrated that zirconia crowns confer superior benefits for gingival health in comparison to stainless steel crowns.^
35
^ Contraindications include periodontal and gingival inflammation, as well as excessive dental arch crowding.^
11
^ To preserve and restore teeth with subgingival defects, several treatment approaches are utilized, including deep marginal elevation, surgical crown lengthening, and orthodontic traction.^
26,70,159
^


### Post-and-Core Restorations

The wide root canal systems of young permanent teeth can be accommodated by modifying fiber posts to achieve a better fit, which helps control adhesive thickness, prevent voids, reduce polymerization shrinkage, and minimize stress transmission to the tooth root.^
13,149
^ There is no specific age limit for permanent post-and-core restorations.^
41
^ For teeth with completed root canals and extensive defects that make direct fillings challenging, fiber posts and resin cores can significantly enhance coronal sealing, reduce microleakage, and improve the long-term prognosis of the restoration.^
137
^ Fiber-reinforced composite posts are known for their biocompatibility, mechanical properties, and aesthetic appeal, and they can be customized to match the root canal morphology.^
83
^ In young permanent teeth that have undergone root canal treatment, minimal preparation of the post space is crucial to maintaining root fracture resistance and preventing irreparable damage.^
113
^ Avoiding excessive post space preparation to maximize dentin preservation constitutes a fundamental principle in contemporary restorative procedures following endodontic therapy.^
75
^ It is preferable to adjust the post according to the existing parameters of the root canal shape, rather than preparing the post space to accommodate a specific post.^
128
^ Inadequate compatibility between the post and root canal parameters can result in an excessively thick or uneven resin cement layer, thereby increasing the risk of porosity inclusion, irregular shrinkage during polymerization, and potential post displacement.^
128
^ Fiber posts help distribute stress more evenly than metal posts, reducing the risk of stress concentration at weak areas of the root neck.^
40
^ In addition, fiber posts are easier to remove if necessary, minimizing the risk of iatrogenic root fractures.^
178
^ While transitional crowns have no age restrictions and can be used throughout development, permanent crown restorations should take into account ongoing growth and development.^
9
^ Early application of all-ceramic restorations is feasible, with the potential for replacement in adulthood while preserving the original fiber posts.^
72,78,164
^ Recent studies have reported that advanced short fiber-reinforced composites (SFRCs) achieve robust chemical bonding between glass fibers and the resin matrix.^
64
^ SFRCs offer both structural and chemical reinforcement to compromised teeth.^
14
^ The intricate architecture and orientation of short fibers, when integrated with the composite resin matrix, enhance the material’s ability to effectively limit crack propagation following the application of mechanical forces.^
87
^ This synergistic combination holds significant potential in preventing fractures in ETT, thereby contributing to the longevity and reliability of restorative treatments.^
22,185
^ It is essential to encapsulate fiber-reinforced composite posts with conventional composites to mitigate the risk of hydrolysis between the fibers and the resin matrix.^
173
^ The elastic modulus of fiber posts closely approximates that of dentin, which may contribute to reducing the likelihood of root fractures.^
161
^ Considering that the mechanical properties of the entire restorative system—including the post, cement, and dentin—must be harmonized, the use of fiber posts bonded with composite resin materials offers a promising approach for achieving optimal clinical outcomes.^
158
^ This composite integration enhances the uniform distribution of stresses, potentially improving the longevity and effectiveness of root canal restorations.^
13
^ Posts primarily provide retention for crown restorations and are suitable for teeth with substantial coronal structure loss, while in cases of irregularly shaped or highly divergent root canals, alternative solutions such as bonded custom fiber posts and fiber bundles can be used.^
162
^


To restore young permanent molars with extensive carious lesions or those that have undergone endodontic treatment-considering factors such as longevity, precision, aesthetics, minimal tooth reduction, and time efficiency, endocrown restorations utilizing a digital workflow and CAD/CAM blocks may represent a novel treatment option.^
2,99,150
^ Another advantage of digital intraoral scanning is its ability to assess the volume of residual tooth structure, encompassing the remaining coronal dentin, the effects of the banding phenomenon, and the residual walls in all dimensions.^
128
^ The supra-gingival margin contributes to better preservation of gingival health and facilitates oral hygiene maintenance, while the use of endocrown demonstrates superior efficacy in restoring short crowns, calcified root canals, and fine roots.^
123
^ The preparation is confined exclusively to the occlusal surface without proximal adjustments to preserve maximal tooth structure, while the custom restoration extended into the pulp chamber to enhance retention.^
46
^ The optimal marginal fit, color stability, and surface condition of root canal crowns are directly associated with proper preparation design, selection of ceramic material, favorable anatomical features provided by the CAD/CAM system, and the type of adhesive used.^
167
^


## Timing of Restorations

Patients undergoing pulp treatment for young permanent teeth face unique challenges related to their age and developmental stage.^
3
^ Regular dental check-ups and oral hygiene counseling play a crucial role in establishing a baseline for oral health needs, facilitating the identification of specific treatment requirements as tooth eruption progresses, and ensuring timely interventions and optimal care.^
15
^ The primary treatment objectives are to preserve the integrity of the dental arch, maintain adequate tooth structure for future restorative procedures, optimize functional restoration, and enhance aesthetic outcomes.^
192
^ The early initiation and sustained provision of appropriate dental care throughout all stages of life are essential for effectively addressing patients’ dental needs and enhancing long-term prognostic outcomes.^
207
^ The selection of minimally invasive restorative treatments necessitates periodic evaluation and maintenance in accordance with the “5 Rs” (Reviewing, Resealing, Refurbishing, Repairing, Replacing) as required, thereby ensuring optimal clinical outcomes and long-term dental health.^
7
^ In children aged 6 to 9 years, the first permanent molars are often not fully developed, and various diseases can hinder apical development, resulting in large apical foramina and shorter roots.^
112
^ To address these issues, endodontic procedures such as pulp revascularization and apexification are employed to encourage continued apical development, often requiring multiple visits.^
142
^ During the treatment period, it is crucial to protect the affected teeth. Although glass ionomer or resin fillings are commonly used for this purpose, the feasibility and protective benefits of preformed crowns during pulp treatment are poorly studied.^
177
^ The open-faced stainless steel crowns have the potential to offer significant protection to first permanent molars during endodontic procedures, with access created through the occlusal surface for subsequent treatments.^
114
^ Nevertheless, more research is needed to evaluate the effectiveness of different sealing materials for occlusal openings, their sealing efficacy, and their impact on patient oral hygiene. For patients aged 10 to 12 years, the first permanent molar roots are usually fully developed, while the second permanent molars and premolars have not yet erupted.^
63
^ In this context, crown restorations for the first permanent molars must be carefully planned to ensure they do not interfere with the eruption of adjacent teeth. The timing of permanent restorations is not universally standardized and depends on several factors, including occlusal relationship stability, changes in crown margin position, and the anatomical characteristics of young permanent teeth.^
120,202
^ Restoration timing considerations include performing permanent restorations around ages 12 to 13 when the permanent dentition is established to support stable occlusal relationships and the eruption of the second permanent molar; delaying restorations until after the eruption of the second permanent molar between ages 13 and 16, when soft and hard tissues are still developing, with the choice between preformed and permanent restorations based on the extent of the tooth defects; or opting for permanent restorations between ages 16 and 18, when occlusal relationships and tissue stability are better established, making it a more favorable period for such procedures.^
59,121,134
^ Continuous tooth eruption leads to the exposure of gingival margins, thereby rendering the modifiability of restorative materials critically important, while the integration of digital technologies may offer unique advantages in pediatric dentistry.^
57
^ Once the clinical crown height and gingival tissue have stabilized and matured, optimal restorative outcomes can be achieved through procedures such as gingival reshaping, crown lengthening, or orthodontic treatment, all of which contribute to improved functional and aesthetic results.^
192
^ Consequently, selecting materials (nanoceramic resin) that are easier to modify rather than those (zirconia) that are more difficult to bond is preferable to accommodate the changes in gingival contour that occur during adolescent growth.^
176
^ Moreover, some studies advocate for immediate permanent restorations following pulp treatment, regardless of age, to enhance form and function, improve crown retention, and increase root fracture resistance.^
182
^ This could be because children’s temporomandibular joints typically adapt well to occlusal changes, which facilitates some self-adjustment after permanent restorations.^
25
^ Maintaining the arch space or integrity during the development of the dental arch and restoring masticatory function.^
191
^


Root preservation is crucial for maintaining alveolar bone fullness, which can be beneficial until implant restoration.^
148
^ The consensus on the earliest timing for oral implants generally advises against placing implants before the completion of maxillofacial growth, typically around 20 years old.^
27,28
^ This is because growth and development can impact the stability and positioning of implants. During the age range from 20 to 25 years, third molar eruption may affect occlusal relationships and necessitate careful consideration of potential complications before proceeding with implant restoration.^
39,67
^ It is essential to communicate these potential issues to patients and plan accordingly to ensure optimal outcomes for implant treatments.^
39
^


## Summary and Outlook

Currently, there is no universal agreement on the restorative protocols for young permanent molars with defects.^
50
^ Given the critical role of the first permanent molar in a child’s dental development, a comprehensive approach that spans from primary prevention to lifelong management is crucial. This management should encompass various stages, including the embryo phase, pre-eruption, eruption, and post-eruption, with tailored interventions for different disease states.^
62
^ Direct restorations provide coronal sealing for defects arising from caries, pulp and periapical diseases, and structural anomalies.^
151
^ Inlays and high inlays are effective for restoring function and maintaining spatial relationships.^
103
^ Preformed crowns serve as transitional restorations, preserving the affected teeth until more permanent solutions, such as full crowns or post-crowns, can be applied.^
175,177
^ Permanent restorations are feasible once root development is complete, with the understanding that replacements may be necessary in adulthood; this should be clearly communicated before treatment begins, with diligent follow-up afterward.^
1
^ A comprehensive understanding of how occlusal factors and parafunctional stresses influence prognosis is essential.^
6
^ In cases where severe inflammation or non-restorable defects are present, extraction may be necessary.^
42
^ Following extraction, space maintenance or orthodontic treatment is important to prevent issues such as space reduction, arch length shortening, or rebuilding a complete dental arch.^
19
^


With advancements in materials and bonding techniques, various treatment options are now available for dental restorations. For mixed and early permanent dentition, digital restorative technologies offer several advantages for young patients, including reduced anxiety, expanded range of materials, improved replicability of restorations, and the ability to streamline workflows for patients undergoing sedation.^
176
^ Ongoing refinement and quantification of research indicators will enable clinicians to develop systematic approaches, allowing for more precise treatment plans tailored to individual patient needs. Direct and indirect restorations are evolving processes, with a preference for minimally invasive methods in initial treatments to preserve healthy tooth structure for future repairs and minimize complications.^
139
^ Given the unique growth and development characteristics of children’s orofacial systems, it is essential to base clinical decisions on evidence-based research and engage in multidisciplinary discussions.^
146
^ This approach will help in determining the most effective diagnostic and therapeutic methods, as well as establishing appropriate standards for treatment.

## References

**Table 1 Table1:** Comprehensive comparison of restorative strategies for compromised first permanent molars in children

Category	Direct glass-ionomer restoration	Direct composite restoration	Composite resin inlays	Ceramic inlays	Preformed metal crowns	All-ceramic crowns	Post-and-core restorations
Materials	Glass-ionomer cement	Resin composites	Indirect composite resin restorations	Lithium disilicate-polymer-Infiltrated ceramic network	Stainless steel	Monolithic zirconia, lithium disilicate	Fiber or metal posts and composite cores
Advantages	Minimally invasive, releases fluoride ions, biocompatibility, reducing operative time, low technique sensitivity	Minimally invasive, preserving tooth structure, ease of retreatment, time efficiency, cost-effectiveness	Abbreviated treatment duration, superior properties, prevents excessive wear on opposing teeth, minimally invasive, biocompatibility	Aesthetic, precise margins, reduce marginal microleakage or marginal gaps, lower failure rate due to recurrent caries, excellent wear resistance, prevents excessive wear on opposing teeth	Cost-effective, space maintainer	Superior aesthetics, superior properties, biocompatibility	Preserves tooth structure,posts provide retention for crown restorations
Disadvantages	Lower compressive and flexural strengths, compromised aesthetic properties, limited antimicrobial efficacy, limited wear resistance	Recurrent caries, marginal discoloration, composite wear, retention failure, fracture of restorations	Long margin lines, high bonding standards, susceptibility to staining and fracture, technique sensitivity	High cost, the removal of additional dental tissue, technique-sensitive	Risks of detachment, crown perforation, marginal adaptation, unaesthetic appearance impaction of an adjacent second permanent molar and potential metal allergies	Technique-sensitive, requires sufficient structure	Root fracture risk, post removal challenges
Indications	Mild to moderate defects	Small pit/fissure caries, MO/DO cavities (>2mm)	Moderate-large defects, MO/DI restorations	Extensive caries, MIH, ETT with MOD cavities	Endodontically treated molars, multi-surface caries	Intact axial walls, MIH cases	Extensive coronal destruction, root-treated teeth
Clinical considerations	Limited wear resistance	Isolation challenges, occlusal adjustment	Gingival margin placement, occlusal compatibility	Subgingival margin management	Occlusal adjustment, eruption guidance	Digital workflow, gingival margin elevation	Post-space customization, adhesive cementation
Long-term performance	97.42% survival rate for a 3-year period;^ 19 ^ 18.6% failure rate for a 6-year period^ 184 ^	10-year durability (60%)^ 117 ^	91% survival rate at 5 years;^ 6 ^ 79.2%-81% survival rate at 10 years^ 69 ^	92-95% survival rate at 5 years^ 138 ^	8.9% failure rate for a 6-year period^ 184 ^	95% success at 5.5 years^ 51 ^	90% survival rate at 5 years^ 86 ^
